# Preparation of Acute Mouse Hippocampal Brain Sections for Transport to a Distant Electrophysiology Laboratory

**DOI:** 10.1002/cpz1.70461

**Published:** 2026-09-22

**Authors:** Sofia Ioanna Nouka, Loukia Katsouri, Jonathan Rudge, John O'Keefe, Francesco Tamagnini

**Affiliations:** ^1^ Reading School of Pharmacy University of Reading Reading United Kingdom; ^2^ Sainsbury Wellcome Centre for Neural Circuits and Behaviour University College London London United Kingdom; ^3^ Centro Studi Biomedici Università degli Studi della Repubblica di San Marino, University of the Republic of San Marino San Marino

**Keywords:** acute slices, electrophysiology, long‐lasting slice preparation, neuronal excitability, synaptic transmission, transport

## Abstract

This article describes a standardized method for preparing and transporting acute mouse hippocampal brain slices suitable for high‐quality single‐cell, CA1, pyramidal cells electrophysiological recordings between laboratories in departments or institutions located at distances up to 3 hr apart. The procedure emphasizes maintaining tissue viability and preserving physiological integrity during transport. Slices are prepared in sucrose‐based protective solutions under ice‐cold, oxygenated conditions, followed by a controlled rewarming period to restore metabolic activity. For transport, slices are submerged in oxygenated artificial cerebrospinal fluid (aCSF) contained in 250‐ml parafilm‐sealed Duran bottles, ensuring prolonged viability at near‐ambient temperature. The method includes parameters for slice thickness, temperature control, oxygenation duration, and maximum transport time to ensure reproducibility across sites. Validation using patch‐clamp recordings confirms that electrophysiological properties, such as resting membrane potential, input resistance, action potential firing, and spontaneous synaptic neurotransmission, remain comparable to those in freshly prepared slices. This protocol enables collaborative research where brain slice preparation and electrophysiological recordings occur in separate laboratories, while preserving the reliability and quality of single‐cell functional data. This is particularly useful for laboratories located away from animal housing units. © 2026 The Author(s). *Current Protocols* published by Wiley Periodicals LLC.

**Basic Protocol**: Acute brain slice preparation available for transport

## INTRODUCTION

The intricate biochemical processes regulating neuronal signaling, including synaptic transmission, membrane excitability, and ion channel function, require ex vivo models that preserve the native tissue microenvironment and physiological integrity (Perumal & Sah, 2022; Ting et al., 2018). Acute brain slice preparations have become a cornerstone in neurobiology, providing the essential framework for single‐cell electrophysiological investigations into synaptic function, disease mechanisms, and pharmacological modulation (Tamagnini, Novelia, et al., 2015; Tamagnini et al., 2017). However, traditional protocols often limit experiments to a single laboratory, as maintaining slice viability during transport is challenging; this restricts collaborative, multi‐site studies, and compromises reproducibility (Perumal & Sah, 2022). In fact, while transportation methods for acutely sliced human brain tissue have been proposed (Mittermaier et al., 2024; Qi et al., 2026), they are relatively difficult to adopt as they require constant carbogen perfusion or the development of complex carriers which make them logistically challenging.

Recent innovations including N‐methyl‐d‐glucamine (NMDG)‐based protective recovery solutions and advanced oxygenation strategies, have significantly improved the preservation of tissue health and synaptic connectivity in acute slices, even in aged or diseased models (Ting et al., 2018). Building on these theoretical and technical advances, electrophysiological methods have evolved to enable comprehensive intracellular whole‐cell patch‐clamp as well as single‐ and multi‐electrode array extracellular recordings across a variety of applications. These include measuring the temporal and spatial properties of synaptic transmission across local networks, using for example multi‐electrode arrays (Hanna et al., 2017, 2020), and analyzing action potential kinetics, electrotonic membrane properties as well as evaluating pharmacodynamic responses in physiological conditions or in models of human pathology using whole‐cell recordings (Tamagnini, Novelia, et al., 2015; Tamagnini et al., 2017). The Basic Protocol presented here introduces a standardized, low‐cost workflow for preparing and transporting acute brain slices between departments or institutions, ensuring that high‐fidelity single‐cell data, such as measurements of resting membrane potential, input resistance, spike characteristics, and synaptic events, remain robust following transfer. As a proof of principle, this protocol has been tested in 15‐ to 20‐month‐old aged mice and in both wild‐type and knock‐in mice carrying a human gene with mutations associated with familial Alzheimer's disease (AD), the *App* ^NL‐G‐F^ model (Brady et al., 2023; Saito et al., 2014).

This method offers key advantages, including increased experimental flexibility, enhanced opportunities for cross‐departmental collaboration, and improved reproducibility of electrophysiological data. Nonetheless, it also presents certain challenges, such as logistical complexity in maintaining optimal conditions during transport and the risk of reduced cell viability over extended periods, though careful optimization of the protocol will mitigate these drawbacks. Overall, compared to in situ perfusion or organotypic culture methods, transportable acute slices strike a balance between physiological relevance and experimental practicality (Perumal & Sah, 2022).

The protocol yields detailed quantitative data encompassing neuronal and synaptic membrane properties, the frequency and amplitude of spontaneous events, and pharmacological profiles, with applications extending from basic research to translational studies in neurodegenerative models. In summary, the transportable brain slice protocol provides a validated, flexible workflow addressing prior limitations, enabling researchers to acquire high‐quality single‐cell electrophysiological data beyond traditional lab boundaries.

*NOTE*: The protocol here described is conducted on acutely excised tissue, harvested from mice. Ensure that all procedures for humane sacrifice of the animal and tissue extraction are conducted in accordance with the ethical guidelines, Health and Safety regulations, and institutional and national policies applicable in your setting.

## ACUTE BRAIN SLICE PREPARATION AVAILABLE FOR TRANSPORT

This protocol describes preparing acute brain slices for transport between laboratories in distant buildings or institutions. The slices will be prepared in one facility, while the electrophysiological recordings will be performed in another one.

### Materials

Animals
  For this protocol, you can use any mouse strain and age. As an example, in this study, we used a genetically modified (knock‐in) mouse model of β‐amyloid pathology, the App^NL‐G‐F^ mouse (Brady et al., 2023; Saito et al., 2014). The data shown here were obtained from old female homozygous App^NL‐G‐F^ mice between 15 and 20 months of age, and their wild‐type littermates (control mice). The animals were sacrificed and the slices harvested at the Sainsbury Wellcome Centre, University College London in London and transported by train or car to the Pharmacology Department of the University of Reading School of Pharmacy, where the recordings were made. Because of the reduced viability of brain tissue in advanced age, we assume this protocol will also work in younger animals. Throughout, we describe potential variations in addition to our own preferred methods.
Sectioning solution (see recipe)
Carbogen mixture (95% O_2_, 5% CO_2_)
Artificial cerebrospinal fluid (aCSF) (see recipe in Table 1)
Intracellular solutions (see recipe in Table 2)

Surgical tools:
  Scalpel (large belly blade preferred)
  Surgical spring scissors
  Surgical tweezers
  Small and larger bent spatulas
Ice bucket and ice
Filter paper
Cutting matrix (optional)
Water bath, 37°C
250‐ml Duran bottle for transport
Parafilm
Beaker with an adapted slice holder
Modified Pasteur pipette for slices

Additional reagents and materials to section and subsequently record single‐cell patch‐clamp measurements (see “Slicing and recording setup” below)

**Table 1:** An Example of Cryoprotective, Sectioning Solution and Artificial Cerebrospinal Fluid*^a^*

Sectioning solution			Artificial cerebrospinal fluid		
Molecule	[Final] (mM)	Molecular weight (g/mol)	Molecule	[Final] (mM)	Molecular weight (g/mol)
Sucrose	189	342.30	NaCl	124	58.44
Glucose	10	180.16	KCl	3	74.55
NaHCO_3_	26	84.01	NaHCO_3_	24	84.01
KCl	3	74.55	NaH_2_PO_4_	1.25	137.99
MgSO_4_·7H_2_O	5	246.48	MgSO_4_·7H_2_O	1	246.48
CaCl_2_	0.1	110.98	CaCl_2_	2	110.98
NaH_2_PO_4_	1.25	137.99	Glucose	10	180.16

*^a^* Note that in the sectioning solution, in comparison with aCSF, the sucrose replaces NaCl (to prevent the formation of ice crystals), the [Mg^2+^] is higher and the [Ca^2+^] is lower (to reduce NMDA‐R dependent excitotoxicity).

**Table 2:** Example of KGluconate‐Based Internal Solutions With and Without Biocytin*^a^*

With Biocytin			Without Biocytin		
Molecule	[Final] (mM)	Molecular weight (g/mol)	Molecule	[Final] (mM)	Molecular weight (g/mol)
KGluconate	145	234.2	KGluconate	145	234.2
NaCl	5	58.44	NaCl	5	58.44
HEPES free acid	10	238.3	HEPES free acid	10	238.3
EGTA	0.2	380	EGTA	0.2	380
GTP Na salt	0.3	523	GTP Na salt	0.3	523
ATP Mg salt	4	507	ATP Mg salt	4	507
‐	‐	‐	Biocytin	13.4	372

*^a^* Note that, to make space to the intracellular marker biocytin and keep the osmolarity constant, the concentration of KGluconate has been decreased in the “without biocytin”, in comparison to “with biocytin.”

#### Slicing and recording setup

To section and subsequently record single‐cell patch‐clamp measurements, you will need the following setup.

##### Vibratome

A vibrating microtome, capable of maintaining the sectioning solution at ice‐cold temperatures, is required for tissue sectioning. For example, Leica VT1200 or Campden 9000SMZ vibratome.

###### Microscope

For visualizing cells in brain slices, you will need a fixed‐stage, upright microscope, mounted on a translation table. To better visualize the cells within the brain parenchyma, we recommend using infrared differential interference contrast (IR‐DIC). Epifluorescence or confocal (1‐ or 2‐photon based) microscopy can also be used to visualize specific neuronal subpopulations or patched neurons filled with a fluorescent probe; however, this is optional.

###### Perfusion system

During the recordings, the slices must be continuously perfused with carbogen‐bubbled, heated (35°C) aCSF. To achieve this, place the slices in a submersion‐style recording chamber, with aCSF delivered and removed by a pump system or by gravity.

###### Micromanipulator

Use a motorized or hydraulic or piezoelectric micromanipulator, with low drift and sub‐micron resolution. This will be used to mount the amplifiers head‐stage and guide the micropipette to the cell.

###### Isolation table

The whole setup should be mounted on an anti‐vibration table. This increases the stability of the patch and decreases the probability of losing the seal. The anti‐vibration table can be an active control air table or a passive control one (i.e., a heavy metallic plate placed onto inflated tires or inner tubes).

###### Amplifier

We recommend using an amplifier designed for patch‐clamp recordings (e.g., Molecular Devices, HEKA, etc.). The amplifier is connected to a head‐stage, which is mounted directly on the micromanipulator. Patch‐clamp amplifier head‐stages are designed to be connected both to an AgCl electrode via a micropipette holder and to an Ag/AgCl reference electrode placed in the recording chamber. The pipette holder allows a micropipette filled with internal solution to be firmly placed over the recording electrode; the Ag/AgCl will act as a bridge between the metallic circuitry within the amplifier (where electrons are the charge‐bearing particles) and the solutions (where ions are the charge‐bearing particles).

###### Analog/Digital (A/D) interface

This is needed to convert the amplified analogic signal into a digital signal that can be visualized with dedicated data acquisition software, such as pClamp (Molecular Devices), PatchMaster (HEKA), or Signal (Cambridge Electronic Design). A/D boards specifically designed for electrophysiological recordings include the Digidata 1550 (and previous versions), from Molecular Devices and several adaptable boards from National Instruments.

###### Glass micropipettes

These pipettes can be purchased already pulled and polished. However, most electrophysiology laboratories pull them from borosilicate capillaries using a horizontal/vertical puller. Narishige is one of the most common manufacturers of these pullers. A Narishige micro‐forge can be purchased separately to round off the sharp edges of the pulled pipette. After pulling and polishing, the pipette tip diameter should be ∼1 µm, corresponding to a pipette access resistance of 2 to 7 MΩ.

###### Faraday cage

A Faraday cage should be placed around the recording system and every device directly connected to AC current should be left outside of it. Reducing noise via adequate shielding, cleanliness, and minimizing contact with the space outside the cage (e.g., by introducing a dripper in the inlet perfusion if the reservoir is placed outside the cage) is always preferable to filtering and active noise control. The latter approaches can lead to data loss or, in some cases, even add noise.

###### Temperature control

Temperature control within the recording chamber is of paramount importance, as temperature has a dramatic effect on the electrical properties of the plasma membrane. An active temperature control system with constant feedback (i.e., Scientifica Temp Controller) is advisable. However, a constant current system is also adequate if room temperature conditions remain stable.

#### Overview

**Figure 1:**
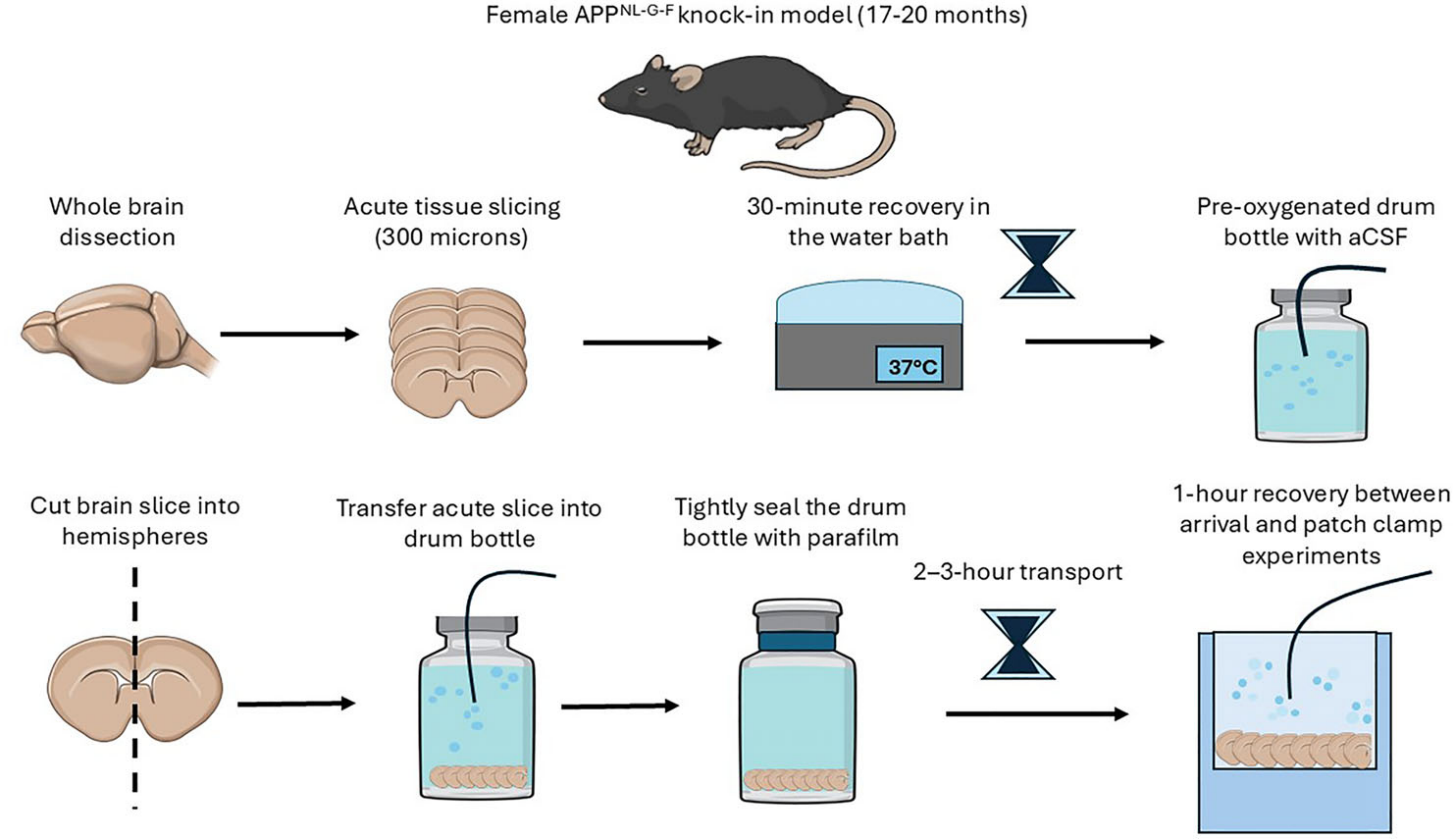
Steps for preparing mouse brain slices suitable for transport between departments and institutions. This protocol closely follows most published methods for preparing brain slices for single‐cell electrophysiological recordings. The key difference is that, after recovery from slicing, the slices are placed in a 250‐ml Duran bottle containing oxygenated aCSF. Under these conditions, the slices remain viable for up to 4 hr (and possibly longer, although this was not assessed), supporting high‐quality single‐cell electrophysiological recordings using whole‐cell patch clamp.

Brain slicing followed by manual patch‐clamp techniques is known to be challenging. Preparing slices for transport is not substantially different from any other established paradigms for acute slicing and is based on using sealed vessels to prevent a decrease in oxygen (O_2_) partial pressure in the aCSF during transportation. In fact, the main cause of cell death and slice deterioration is arguably insufficient oxygenation. To avoid using portable carbogen bubbling setups with open vessels for transport, we opt for a more cost‐effective and intuitive solution: bubbling the transport aCSF at room temperature in a 250‐ml Duran bottle and, after placing the slices in it, closing it very tightly and wrapping the cap in parafilm to minimize the loss of O_2_ partial pressure. The rationale for this approach assumes that the O_2_ dissolved in 250 ml of aCSF is more than sufficient to sustain *n* = 6 to 10, 300‐µm mouse brain slices for a duration of 2 to 3 hr during transport. Figure 1 summarizes the necessary steps in this protocol.

#### Slice preparation and transport

The following section will outline the sequence of operations which will be conducted in the two institutions, respectively. Institution 1 will be where the slices will be prepared, Institution 2 where the recordings will be conducted.

##### Steps to be conducted at Institution 1

**Figure 2:**
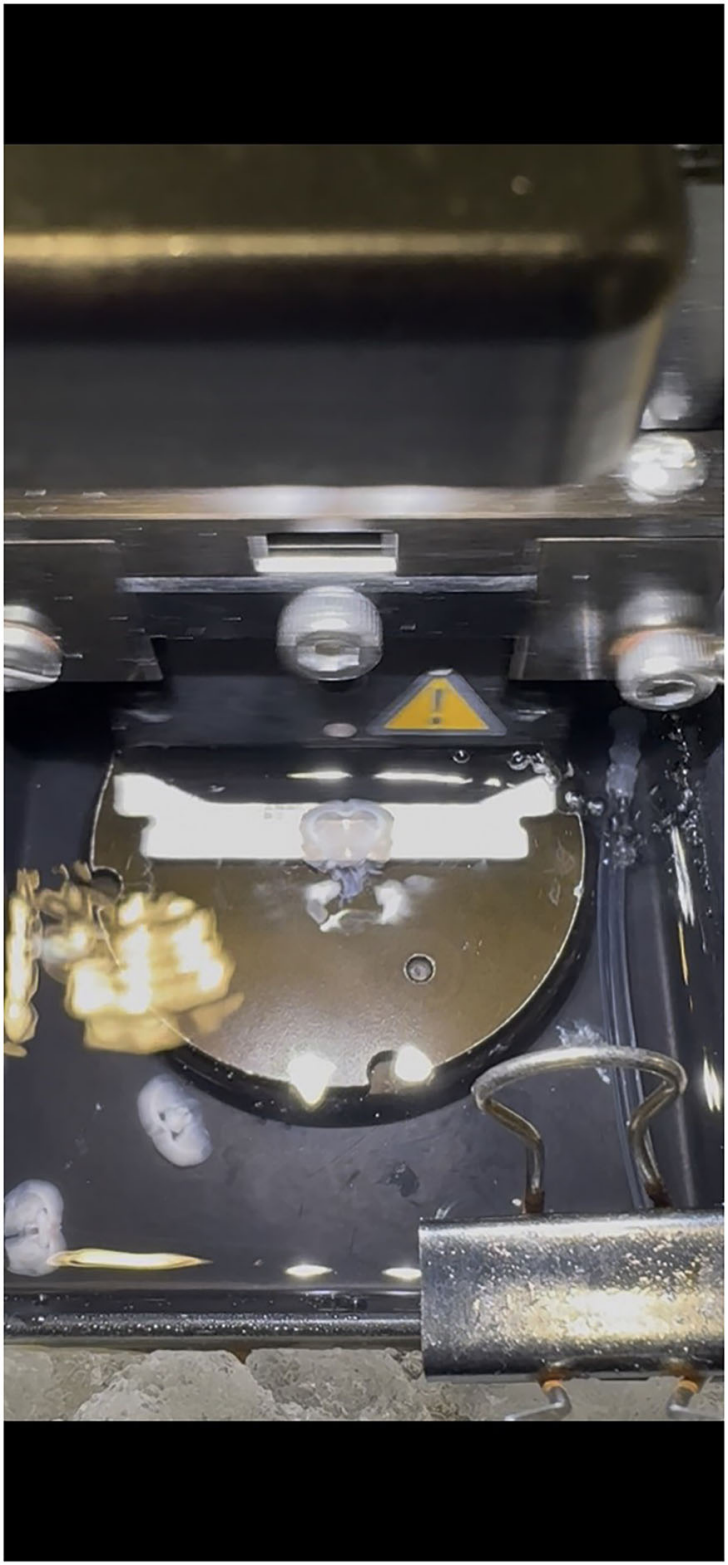
Acute brain sections can be obtained using a vibration microtome.

1 Euthanasia. Kill and decapitate the animal following cervical dislocation.
2 Brain excision. Rapidly remove the brain using surgical tools and place into an ice‐cold (0° to 4°C) cryoprotective sectioning solution, which is continuously bubbled with carbogen.
3 Brain dissection. Depending on the brain area of interest, remove specific regions with single scalpel cuts after placing the brain on filter paper on a chilled surface.
  For example, if the prefrontal cortex is required, the cerebellum and the caudal part of the telencephalon can be removed with single scalpel cuts. Slice were sectioned at 300 µm in this paradigm.
4 Matrix. To improve the accuracy and the precision of these cuts, use a brain cutting matrix (e.g., the matrix for coronal cuts of mouse brains from WPI instruments, cat no. RBMS‐205C).
5 Brain mounting. Depending on the required anatomical plane, glue the brain to the vibratome stage in different orientations.
  For example, to cut horizontal slices containing the striatum, hippocampus, and temporal cortices, you should remove the cerebellum, the prefrontal lobes, and the dorsal part of the cortex with single scalpel cuts, then glue the brain on its dorsal side (ventral side facing up).
6 Sectioning. Quickly place the sample into the vibratome tray, covered with ice‐cold, carbogen‐bubbled sectioning solution, and cut the slices (Fig. 2).
  Critical passage. The thickness of the slice can range between 250 and 500 µm. While 400 µm is commonly the preferred thickness for field potential or blind patch‐clamp recordings, for visualized patch‐clamp using infrared DIC microscopy, we recommend not exceeding a thickness of 300 µm.

**Figure 3:**
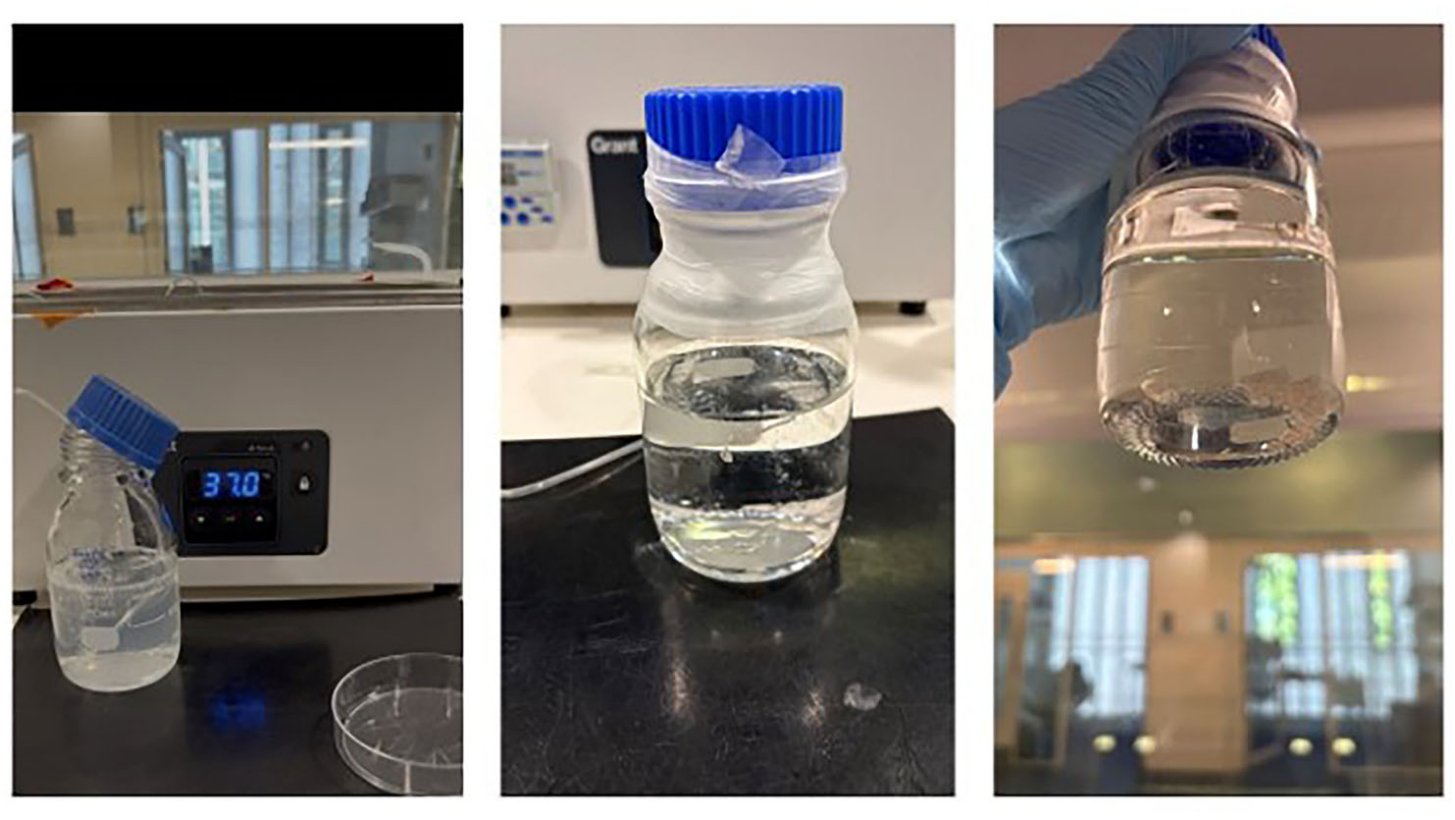
Pre‐oxygenating aCSF with carbogen (left), enables the transport of acute slices in 250‐ml Duran bottles, if the bottles are well sealed (center and right). Sealing with Parafilm helps maintaining the partial pressures of O_2_ and CO_2_ in the transport solution.

7 Recovery. Immediately place each slice in carbogen‐bubbled aCSF to recover before packing and transport.
  Critical passage. The recovery period should be conducted for 30 minutes at 37°C, by placing the vessel containing the slices in carbogen‐bubbled aCSF in a heated water bath. After this boost phase, the slices are left to recover for 1 hr in bubbled aCSF at room temperature.
8 Transport from Institution 1 to Institution 2. Keep aCSF continuously bubbled in a separate 250‐ml Duran bottle at room temperature (25°C) until the very last moment before closing it. The bottle will be filled to the top (250 ml) with freshly oxygenated (for at least 1 hr) aCSF. After placing the slices in the bottle, close it with its cap and tightly wrap the edge between the cap and the glass bottle with Parafilm (Fig. 3).
  Up to 12 slices at a time were transported. No temperature control was used (the bottle was just wrapped in a plastic bag to avoid potential spillage and then placed in a carrier bag). Oxygen levels and pH were not monitored.
  Critical passage. The bottle should be filled at least halfway or up to the top, and the bubbler should be positioned at the midpoint of the bottle's height. Make sure you place the slices gently at the bottom so that they are not disturbed by the constant bubbling. After sealing the bottle, the slices can be kept in it for up to 3 hr. They may last longer, but we did not test this. Be mindful during transport to avoid excessive shaking of the bottle as this may damage the slices.

###### Steps to be conducted at Institution 2

**Figure 4:**
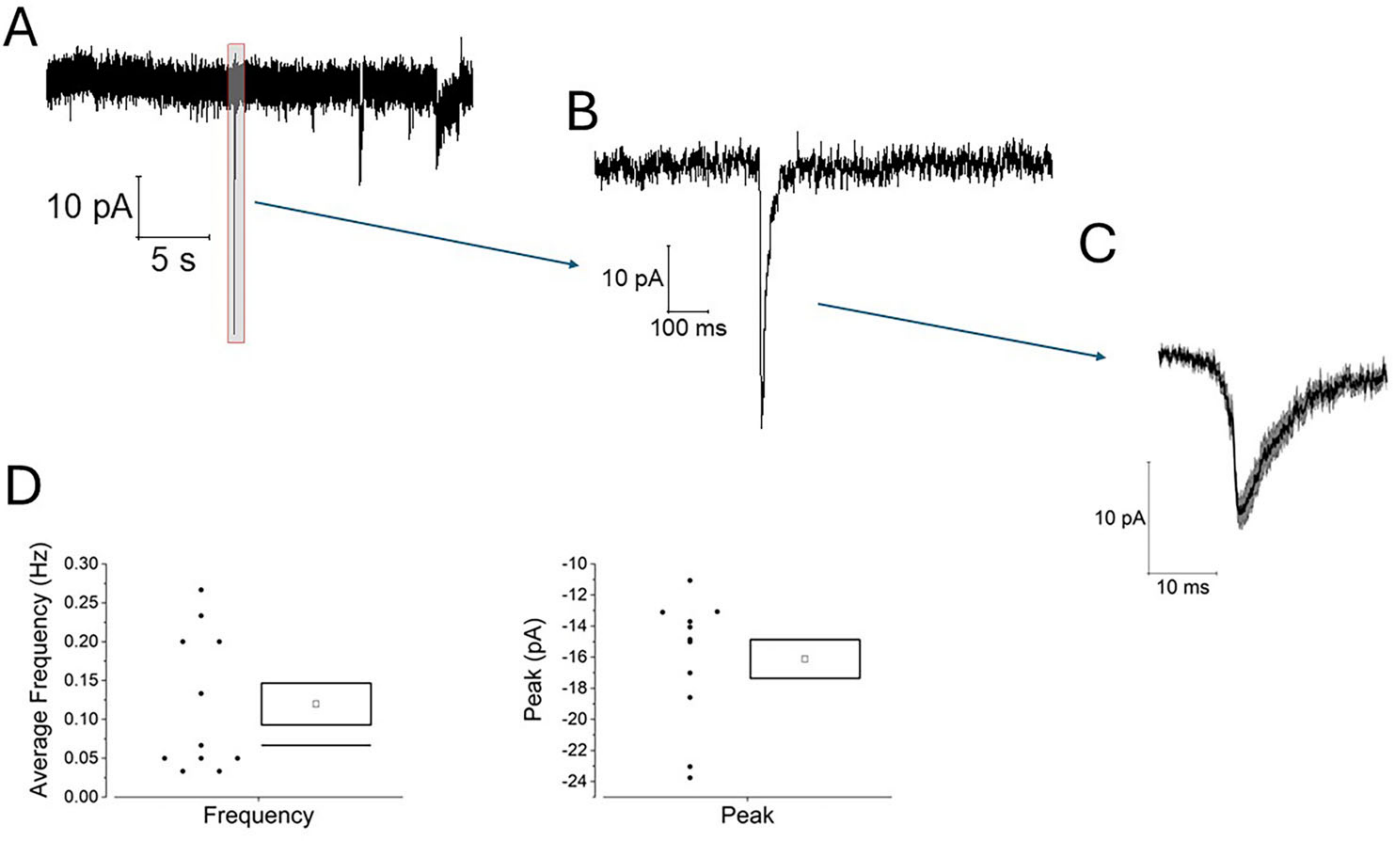
Acute brain sections transported for 2 to 3 hr between institutions retain hippocampal CA1 synaptic activity. (**A**) Representative trace of a gap‐free, voltage‐clamp, patch clamp recording from a CA1 pyramidal neuron in a 17‐month‐old *App* ^NL‐G‐F^ wild‐type mouse. Spontaneous excitatory post‐synaptic currents (sEPSCs) are visible in this trace. The red‐outlined, greyed‐out box magnified in (**B**), shows a clean, isolated sEPSC of >40 pA indicating that the slice preserves an intact, synaptically active local network. (**C**) Average ± *SEM* boundaries of the sEPSCs observed in *n* = 11 wild‐type CA1 PCs obtained from transported hippocampal slices. (**D**) Cell‐to‐cell analysis reporting the single cell's sEPSC frequency (left) and peak amplitude (right). The center of the box represents the average, the single line the median and the top/bottom sides of the box the *SEM* boundaries. Same for all the subsequent scatter box plots.

**Figure 5:**
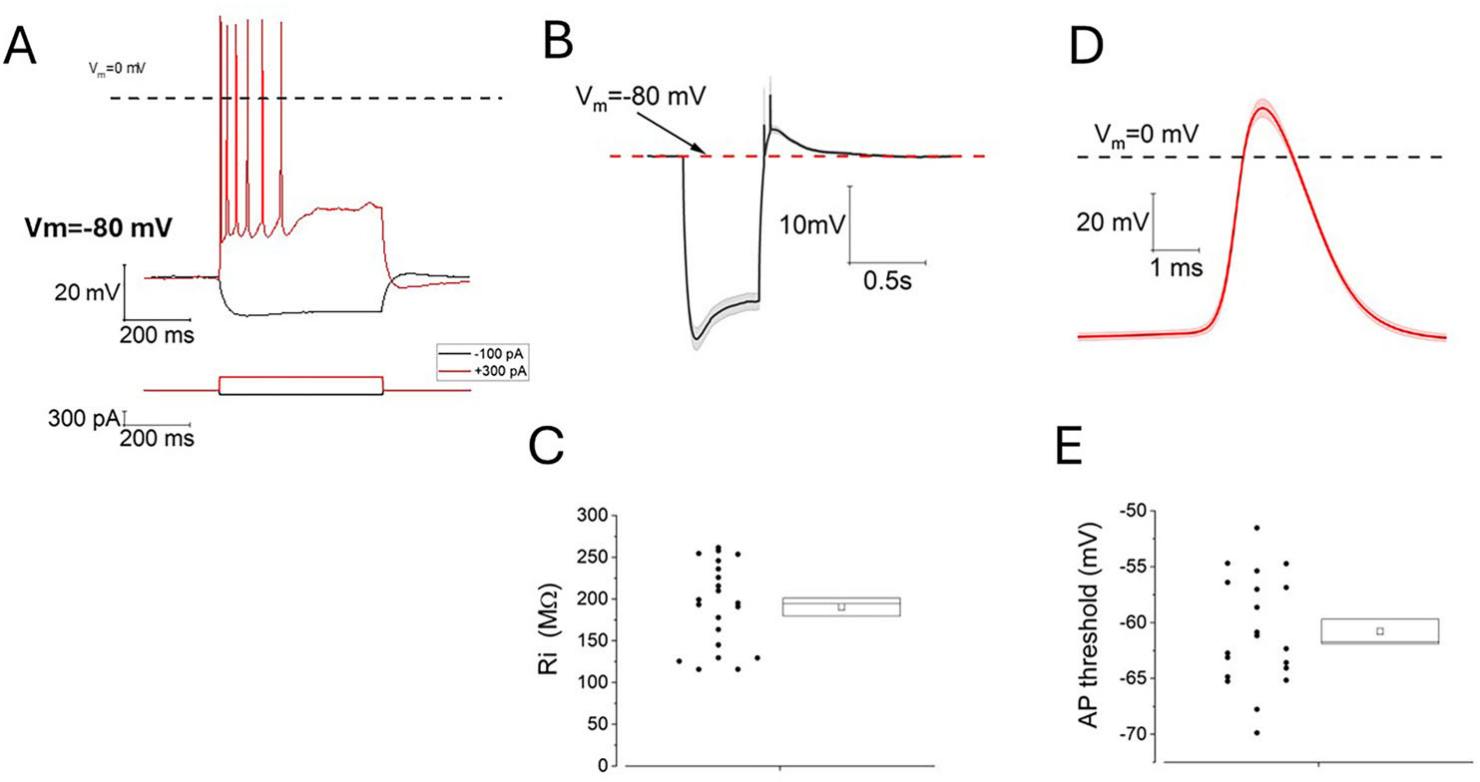
Acute brain sections transported for 2 to 3 hr between institutions retain hippocampal CA1 excitability properties. (**A**) Example traces of a current‐clamp recording obtained from a CA1 PC following 3‐hr transportation. The V_m_ was maintained at a pre‐stimulus value of –80 mV thanks to a constant current injection (I_m_). The black trace in the bottom panel represents a –100 pA, 500 ms current injection, evoking the V_m_ negative deflection observed in the panel above. This was used to measure electrotonic membrane properties, such as the input resistance (Ri). The red trace in the panel below represents a 300 pA, 500 ms square current which leads to evoking the depolarization of the V_m_ and subsequent neuronal firing of action potentials (AP). (**B**) Mean ± *SEM* boundaries trace of the negative V_m_ deflection induced by the –100 pA, 500 ms square I_m_ (*n* = 22) in wild‐type *App* ^NL‐G‐F^ mice. (**C**) Cell‐to‐cell analysis of the input resistance, calculated as V_m_ at the steady state of the deflection divided by the I_m_ (*–*100 pA). (**D**) Mean ± *SEM* of the V_m_ during the firing of the first AP evoked by a 300 pA, 500 ms square I_m_. This waveform is often used to measure the properties of the AP, such as its rate of rise, threshold, width, etc. (**E**) Cell‐to‐cell analysis of the AP threshold of the first AP represented in (**D**), defined as the V_m_ at which the dVdt exceeds the value of 20 V/s.

**Figure 6:**
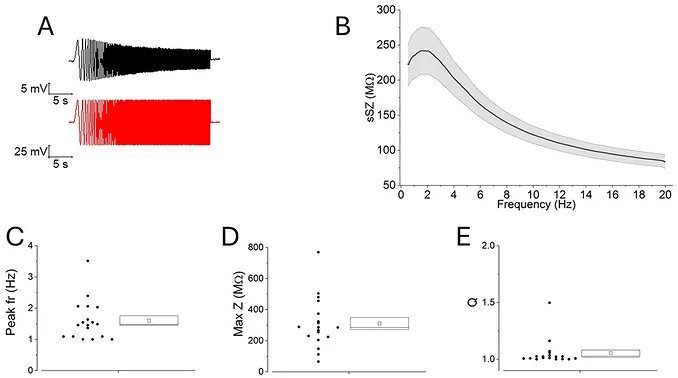
Acute brain sections transported for 2 to 3 hr between institutions retain hippocampal CA1 resonance properties. (**A**) Example traces of injected oscillating current (bottom panel) with a sub‐threshold intensity of ± 50 pA following a 2‐ to 3‐hr transportation. The V_m_ was maintained at a pre‐stimulus value of –80 mV thanks to a constant current injection. (**B**) Mean ± *SEM* boundaries of the smoothed impedance calculated as sSZ = fast Fourier transform (FFT) of the V_m_/FFT(I_m_). The smoothing was obtained by a moving average every 50 datapoints. This is the average of *n* = 18 recordings from wild‐type slices. (**C**‐**E**) Cell‐to‐cell analysis of the resonance properties, such as the Peak frequency (**C**), the Max impedance Z (**D**) and the quality factor of the resonator (calculated as described in Tamagnini et al., 2014) (**E**).

**Figure 7:**
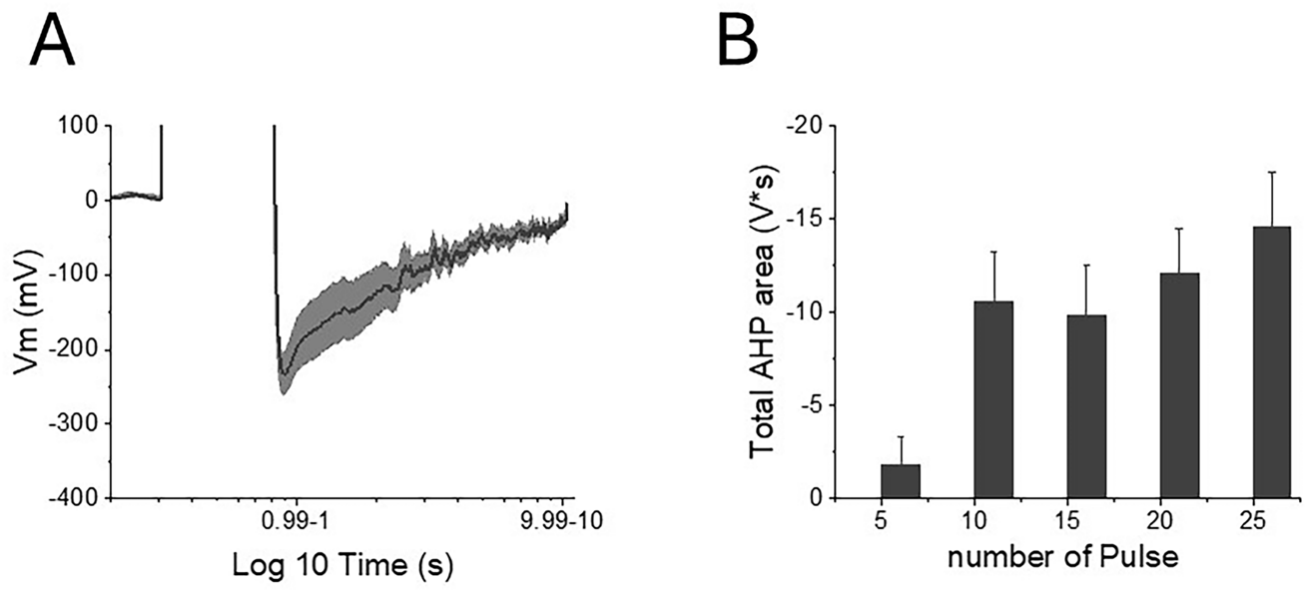
Acute brain sections transported for 2 to 3 hr between institutions retain hippocampal CA1 post‐firing properties. (**A**) Mean ± *SEM* trace of the after hyperpolarization (AHP) evoked by 25 APs delivered at 50 Hz, (each AP evoked by a 2 pA, 2 ms I_m_). Both the medium component of the AHP (within 500 ms from the end of the train) and the slow component are observable. This average has been obtained from wild‐type CA1 PCs (*n* = 12) from a pre‐stimulus V_m_ = –74 mV. (**B**) Histogram showing average ± *SEM* total AHP area as function of the number of pulses evoked.

9 Unpacking the slices. Upon arrival at the destination lab, before opening the transport bottle, ensure you have fresh aCSF continuously bubbled for at least 15 min in a beaker containing a mesh slice holder (or your holder of preference). Only at that point open the transport bottle and gently transfer the slices into the slice holder, preferably using a slice pipette, rather than a paintbrush.
  Critical passage. Before starting the recordings, the slices should be left resting for at least 60 min in carbogen bubbled aCSF at room temperature.
10 Electrophysiology recordings.
  a. Place the slice in the recording chamber and make sure it is stable. Using a harp to hold it down can help.
  b. Identify the area of interest. Use the 4× objective to select the brain area of choice.
  c. Identify the cell of interest. Once the iris has been closed on the area of interest, switch to the high magnification, salt‐water dipping, long‐working‐distance objective (usually 40× or 60×).
  d. Assess the cell's appearance. Use the focus to select a cell that looks healthy. In adult slices, a healthy cell usually appears plump, with smooth regular borders.
  e. Pipette placement and testing. Fill the pipette halfway with the intracellular solution of choice, mount it on the pipette‐holder and position it just above the slice surface. Apply and lock positive pressure inside the mounted pipette. Monitor the pipette electrical resistance by continuously applying small pulses of voltage difference between the recording and the reference electrode (1 to 5 mV). These pulses generate a current with an amplitude inversely correlated to the access resistance of the electrode (Ohm's law: V = RI).
  f. Approach the cell. Using an approach angle on the micromanipulator and the focus, bring the pipette tip toward the cell surface. As you are near the plasma membrane, the access resistance of the electrode should increase, and a dimple should form on the membrane surface. Immediately release the positive pressure at this point.
  g. Obtain the gigaohm seal. Quickly switch on the voltage clamping and set a V_hold_ = –70 mV. Run a protocol enabling the transient passage of V_hold_ from –70 mV to –65 mV. The overall injected current and the current step between V_hold_ = –70 mV and V_hold_ = –65 mV will indicate the tightness of the seal.
  h. Let the cell stabilize. After entering whole‐cell, allow the cell to equilibrate for 1 to 2 min. Monitor the holding current (I_m_) required to maintain the V_hold_ and ensure it remains stable.
  i. Start the experiment. Run the desired protocols to record the electrotonic, electrogenic and synaptic (e.g., EPSCs) properties of the patched cell. Figures 4, 5, 6 to 7 show example traces and cell‐to‐cell analysis of voltage‐ and current‐clamp recordings.

## Reagents and Solutions

### Artificial cerebrospinal fluid (aCSF)

The composition of the cerebrospinal fluid changes slightly from one group to another, but some characteristics are common. (1) The aCSF has a NaHCO_3_‐based buffer system and for this reason it needs to be constantly bubbled with Carbogen. (2) It contains high concentrations of NaCl (usually >100 mM) and low concentrations of KCl (usually <5 mM). (3) The relative concentrations of Ca^2+^ and Mg^2+^ can change (2:1 is common) and they are commonly ≤2 mM. (4) Glucose levels can vary between 1 and 10 mM. See Table 1 for the aCSF formula we use in our laboratory. The pH of the aCSF must be 7.4 and the osmolarity = ∼300 mOsm/L.

### Intracellular solutions

Intracellular solutions can vary, depending on the type of current(s) to be measured. For example, to record outward K^+^ currents starting from a hyperpolarized holding potential (V_hold_) of –100 mV, a high K^+^ and low Cl^–^ internal solution is recommended. In our laboratory, we normally use K‐gluconate‐based internal solutions. However, other counter‐ions for K^+^ can be used, such as methane sulfonate (KMeSO_4_) or Cl^–^. The pH should be 7.4. The osmolarity of the internal solution should be 5% lower than that of the extracellular solution, to avoid cell swelling and weakening or loss of the seal (280 to 290 mOsm/L). If an intracellular tracer will be added to the internal solution (e.g., biocytin for post‐hoc reconstruction), the concentration of the K^+^ salt should be reduced accordingly, to maintain constant osmolarity. See Table 2 for an example of an internal solution used in our laboratory, with and without biocytin.

### Sectioning solution

To minimize hypoxia‐induced damage, all cutting operations are performed at ice‐cold temperatures (0° to 4°C), between decapitation and brain slicing. To further protect the tissue from both hypoxia and cold temperatures, specialized sectioning solutions are used. These solutions typically contain low Ca^2+^ and high Mg^2+^ to limit NMDA receptor‐mediated excitotoxicity. In addition, sucrose or glycerol can replace equimolar NaCl to reduce nucleation of extracellular ice crystals that could damage the plasma membrane. In our experiments, we use a sucrose‐based solution, in which the sucrose acts as a cryoprotectant, and low Ca^2+^ and high Mg^2+^ to prevent excitotoxicity (Table 1). Some laboratories instead use artificial cerebrospinal fluid (aCSF) as a cutting solution. Regardless of composition, these solutions rely on a NaHCO_3_ buffer system and therefore must be continuously bubbled with a gas mixture containing 95% O_2_ and 5% CO_2_ (carbogen) to maintain pH. Choose a sectioning solution and maintain it consistently throughout the entire project.

## Commentary

### Critical Parameters

This protocol is very robust and is based on many years of experience that innumerable laboratories (including ours) have in obtaining, maintaining, and using acute brain slices from mice for the recording of single cell and local network neuronal activity. It is not necessary to use our specific sectioning solution or aCSF, nor to use animals of the same age and genetic background. We also assume that this protocol can be used for other species, including other rodents, primates, and humans.

The most crucial factor for ensuring the maintenance of the slices during transport is that the partial pressures of O_2_ and CO_2_ remain constant. For this reason, the experimenter must ensure that the aCSF is continuously bubbled for at least 10 to 15 min before closing the cap of the transport vessel. Make sure the bottle is in good condition and the cap is new. Use Parafilm at the connection between the cap and the bottle after it has been closed. Avoid shaking the bottle during transport but note that the slices will remain healthy under normal levels of movement during transport (e.g., walking while keeping the bottle in your bag or normal train movements).

### Troubleshooting

See Table 3 for a troubleshooting guide for patch‐clamp recordings on slices transported for up to 3 hr in a sealed container.

**Table 3:** Troubleshooting Guide for Patch‐clamp Recordings on Slices Transported for up to 3 hr in a Sealed Container

Problem	Possible cause	Solution
Poor cell viability	Gas leaking out of the bottle	Use a new bottle and cap; add another layer of Parafilm
Poor cell viability	Long dissection times	Decrease the dissection time by training on more complex animals (e.g., older animals have a thicker skull)
Cells die quickly after breaking in	The rig lines are dirty	Clean the rig hydraulics passing 5 ml of 4% HCl and rinsing for few hours (consider leaving the rinsing going overnight)

### Statistical Analysis

#### Measuring robustness

We reported the robustness of the method by measuring the number of successful vs unsuccessful days. A successful day was defined as a day in which we have obtained at least one recording from one of the used protocols, providing metrics relative to at least one excitability or synaptic property of the cell. In addition, we have reported the average ± *SEM* values of the number of cells patched per day. We have managed to get at least one successful recording in CA1 PCs in 18 out of 20 mice in the transported group (90%). In addition, we have quantified the number of successfully patched cells per day: we averagely patched 2.6 ± 0.4 CA1 PCs/day. This count included the days in which patched 0 cells. If we only include the days in which we patched at least 1 CA1 PC, we have patched 3.2 ± 0.4 CA1 PCs/day.

#### Validating the relevance of the CA1 PCs properties measured between transport modes

We have reported the descriptive statistics of synaptic (Fig. 4), excitability (Fig. 5), resonance (Fig. 6), and afterhyperpolarization (Fig. 7), from transported wild‐type slices. No hypothesis testing statistical test was carried out as the aim of this article is not to investigate the biological effect of transportation over excitability and synaptic properties of CA1 PCs, but to demonstrate that transportation allows the preparation to be viable for the recording of these properties.

### Understanding Results

We have used this protocol to perform single‐cell electrophysiological recordings in *n* = 10 wild‐type and *n* = 10 *App* ^NL‐G‐F^ female mice, between 15 and 20 months of age. We successfully obtained high‐quality recordings from >40 CA1 pyramidal neurons. These recordings are part of a larger project, and the specific hypotheses assessed in these experiments are not discussed here, as they are not directly relevant. However, we present several example recordings obtained from one of these cells to characterize its electrotonic, electrogenic, post‐firing, resonance, and synaptic properties.

While the aim of this article is not to quantitatively investigate the effect of transport over excitability and synaptic properties in CA1 PCs, we have decided to report the mean ± *SEM* of excitability and synaptic properties of the transported preparations obtained from wild‐type animals to show that the transported preparation is viable for performing electrophysiological recordings. In fact, testing whether transport has a biological effect, while potentially providing an interesting insight over the sensitivity of the preparation, does not add relevant information for the experimenter. In fact, a significant difference in any of the recorded properties would have not precluded the viability of the preparation, while lack of significant differences would have still not granted the indiscriminate use of transported or freshly made slices without accounting “transport” as a biasing factor in the experimental design.

Synaptic, excitability, resonance, and post‐firing properties distributions comparable to what has been already observed in our and other laboratories (Booth, Ridler, et al., 2016; Booth, Witton, et al., 2016; Brown et al., 2011; Goniotaki et al., 2024; Gu et al., 2005; Hu et al., 2002, 2009; Kerrigan et al., 2014; Tamagnini, Novelia, et al., 2015; Tamagnini, Scullion, et al., 2015; Tamagnini et al., 2017).

In addition, our method showed to be robust, with at least one successful recording obtained in 90% of the mice and a relatively high number of successfully patched cells/day. These results are comparable to what has already been observed for transportable human brain slices, a technique already explored in the recent past (Mittermaier et al., 2024; Qi et al., 2026). The main difference between our work and the ones cited is that we work on animal tissue. This is particularly important because one of the premises of Qi et al. (2026) was that animal tissue does not require to be transported, because animal storage facilities are usually close to the electrophysiology lab. This is a situation which is, in fact, turning, as many bioresource units are closing in the United Kingdom and internationally, potentially forcing many physiology laboratories to stop their work on acute tissue. Another important difference is that Mittermaier et al. (2024) propose a compelling, but hard to manage and potentially expensive, system for the transport of unsliced brain human tissue. On the other hand, while Qi et al. (2026) propose a method of transportation for sliced human brain tissue which involves the use of compressed gas canisters in a moving vehicle. While these methods provide an effective system of transportation, ours is particularly simple and cost‐effective, with a comparable level of robustness. While we do not have data to substantiate the claim, it is plausible that our protocol can be applied to other tissues and to other species, including primates and humans. However, to prove this, future work is required.

To conclude, here we provide a detailed description of a cost‐effective, simple, and effective method for transporting animal brain tissue between laboratories, which is viable for carrying out electrophysiological recordings. This is particularly useful for promoting collaborative work between laboratories and providing a viable way to continue working to those laboratories which are experiencing the closure of their bioresource units.

### Time Considerations

The entire protocol will take 1 day, from morning to evening. The steps from euthanizing the animal to the start of the recordings will take ∼5 hr, assuming a 3‐hr transport time.

### Author Contributions

**Sofia Ioanna Nouka**: Investigation; methodology; validation; visualization; writing—review and editing. **Loukia Katsouri**: Funding acquisition; investigation; methodology; resources; supervision; writing—review and editing. **Jonathan Rudge**: Funding acquisition; investigation; methodology; project administration; resources; supervision; writing—review and editing. **John O'Keefe**: Conceptualization; funding acquisition; investigation; methodology; project administration; resources; supervision; writing—review and editing. **Francesco Tamagnini**: Conceptualization; funding acquisition; investigation; methodology; project administration; resources; supervision; visualization; writing—original draft; writing—review and editing.

### Conflict of Interest

Jonathan Rudge has contributed financially to the realization of this project by partially funding Sofia Ioanna Nouka's PhD research expenses.

## Data Availability

The data, tools, and material (or their source) that support the protocol are available from the corresponding author upon reasonable request.

## References

[cpz170461-bib-0001] Booth, C. A. , Ridler, T. , Murray, T. K. , Ward, M. A. , de Groot, E. , Goodfellow, M. , Phillips, K. G. , Randall, A. D. , & Brown, J. T. (2016). Electrical and network neuronal properties are preferentially disrupted in dorsal, but not ventral, medial entorhinal cortex in a mouse model of tauopathy. The Journal of Neuroscience, 36(2), 312–324. 10.1523/JNEUROSCI.2845-14.2016 PMC4710763 26758825

[cpz170461-bib-0002] Booth, C. A. , Witton, J. , Nowacki, J. , Tsaneva‐Atanasova, K. , Jones, M. W. , Randall, A. D. , & Brown, J. T. (2016). Altered intrinsic pyramidal neuron properties and pathway‐specific synaptic dysfunction underlie aberrant hippocampal network function in a mouse model of tauopathy. The Journal of Neuroscience, 36(2), 350–363. 10.1523/JNEUROSCI.2151-15.2016 PMC4710765 26758828

[cpz170461-bib-0003] Brady, E. S. , Griffiths, J. , Andrianova, L. , Bielska, M. H. , Saito, T. , Saido, T. C. , Randall, A. D. , Tamagnini, F. , Witton, J. , & Craig, M. T. (2023). Alterations to parvalbumin‐expressing interneuron function and associated network oscillations in the hippocampal ‐ medial prefrontal cortex circuit during natural sleep in App(NL‐G‐F/NL‐G‐F) mice. Neurobiology of Disease, 182, 106151. 10.1016/j.nbd.2023.106151 37172910

[cpz170461-bib-0004] Brown, J. T. , Chin, J. , Leiser, S. C. , Pangalos, M. N. , & Randall, A. D. (2011). Altered intrinsic neuronal excitability and reduced Na+ currents in a mouse model of Alzheimer's disease. Neurobiology of Aging, 32(11), 2109.e1–2109.e14. 10.1016/j.neurobiolaging.2011.05.025 21794952

[cpz170461-bib-0005] Goniotaki, D. , Tamagnini, F. , Biasetti, L. , Rumpf, S. L. , Troakes, C. , Pollack, S. J. , Ukwesa, S. , Sun, H. , Kraev, I. , Serpell, L. C. , Noble, W. , Staras, K. , & Hanger, D. P. (2024). Tau‐mediated synaptic dysfunction is coupled with HCN channelopathy. Alzheimers Dement, 20(8), 5629–5646. 10.1002/alz.14074 PMC11350046 38994745

[cpz170461-bib-0006] Gu, N. , Vervaeke, K. , Hu, H. , & Storm, J. F. (2005). Kv7/KCNQ/M and HCN/h, but not KCa2/SK channels, contribute to the somatic medium after‐hyperpolarization and excitability control in CA1 hippocampal pyramidal cells. The Journal of Physiology, 566(Pt 3), 689–715. 10.1113/jphysiol.2005.086835 PMC1464792 15890705

[cpz170461-bib-0007] Hanna, L. , Kawalek, T. J. , Beall, C. , & Ellacott, K. L. J. (2020). Changes in neuronal activity across the mouse ventromedial nucleus of the hypothalamus in response to low glucose: Evaluation using an extracellular multi‐electrode array approach. Journal of Neuroendocrinology, 32(3), e12824. 10.1111/jne.12824 PMC7064989 31880369

[cpz170461-bib-0008] Hanna, L. , Walmsley, L. , Pienaar, A. , Howarth, M. , & Brown, T. M. (2017). Geniculohypothalamic GABAergic projections gate suprachiasmatic nucleus responses to retinal input. The Journal of Physiology, 595(11), 3621–3649. 10.1113/jp273850 PMC5451736 28217893

[cpz170461-bib-0009] Hu, H. , Vervaeke, K. , Graham, L. J. , & Storm, J. F. (2009). Complementary theta resonance filtering by two spatially segregated mechanisms in CA1 hippocampal pyramidal neurons. The Journal of Neuroscience, 29(46), 14472–14483. 10.1523/jneurosci.0187-09.2009 PMC6665813 19923281

[cpz170461-bib-0010] Hu, H. , Vervaeke, K. , & Storm, J. F. (2002). Two forms of electrical resonance at theta frequencies, generated by M‐current, h‐current and persistent Na+ current in rat hippocampal pyramidal cells. The Journal of Physiology, 545(Pt 3), 783–805. 10.1113/jphysiol.2002.029249 PMC2290731 12482886

[cpz170461-bib-0011] Kerrigan, T. L. , Brown, J. T. , & Randall, A. D. (2014). Characterization of altered intrinsic excitability in hippocampal CA1 pyramidal cells of the Abeta‐overproducing PDAPP mouse. Neuropharmacology, 79, 515–524. 10.1016/j.neuropharm.2013.09.004 PMC3989024 24055500

[cpz170461-bib-0012] Mittermaier, F. X. , Alle, H. , & Geiger, J. R. P. (2024). Long‐distance transport of surgically resected, alive human brain tissue. In J. H. R. Lübke & A. Rollenhagen (Eds.), New Aspects in Analyzing the Synaptic Organization of the Brain (pp. 3–17). Springer US. 10.1007/978-1-0716-4019-7_1

[cpz170461-bib-0013] Perumal, M. B. , & Sah, P. (2022). A protocol to investigate cellular and circuit mechanisms generating sharp wave ripple oscillations in rodent basolateral amygdala using ex vivo slices. STAR Protocols, 3(1), 101085. 10.1016/j.xpro.2021.101085 PMC8761775 35072114

[cpz170461-bib-0014] Qi, G. , Yang, D. , Bak, A. , Hucko, W. , Delev, D. , Hamou, H. , Feldmeyer, D. , & Koch, H. (2026). Transport‐related effects on intrinsic and synaptic properties of human cortical neurons: A comparative study. The Journal of Physiology, 604(5), 2060–2082. 10.1113/JP288111 PMC12953011 41609472

[cpz170461-bib-0015] Saito, T. , Matsuba, Y. , Mihira, N. , Takano, J. , Nilsson, P. , Itohara, S. , Iwata, N. , & Saido, T. C. (2014). Single App knock‐in mouse models of Alzheimer's disease. Nature Neuroscience, 17(5), 661–663. 10.1038/nn.3697 24728269

[cpz170461-bib-0016] Tamagnini, F. , Novelia, J. , Kerrigan, T. L. , Brown, J. T. , Tsaneva‐Atanasova, K. , & Randall, A. D. (2015). Altered intrinsic excitability of hippocampal CA1 pyramidal neurons in aged PDAPP mice. Frontiers in Cellular Neuroscience, 9, 372. 10.3389/fncel.2015.00372 PMC4604241 26528126

[cpz170461-bib-0017] Tamagnini, F. , Scullion, S. , Brown, J. T. , & Randall, A. D. (2014). Low concentrations of the solvent dimethyl sulphoxide alter intrinsic excitability properties of cortical and hippocampal pyramidal cells. PLoS One, 9(3), e92557. 10.1371/journal.pone.0092557 PMC3960278 24647720

[cpz170461-bib-0018] Tamagnini, F. , Scullion, S. , Brown, J. T. , & Randall, A. D. (2015). Intrinsic excitability changes induced by acute treatment of hippocampal CA1 pyramidal neurons with exogenous amyloid beta peptide. Hippocampus, 25(7), 786–797. 10.1002/hipo.22403 PMC4791149 25515596

[cpz170461-bib-0019] Tamagnini, F. , Walsh, D. A. , Brown, J. T. , Bondulich, M. K. , Hanger, D. P. , & Randall, A. D. (2017). Hippocampal neurophysiology is modified by a disease‐associated C‐terminal fragment of tau protein. Neurobiology of Aging, 60, 44–56. 10.1016/j.neurobiolaging.2017.07.005 PMC5654728 28917666

[cpz170461-bib-0020] Ting, J. T. , Lee, B. R. , Chong, P. , Soler‐Llavina, G. , Cobbs, C. , Koch, C. , Zeng, H. , & Lein, E. (2018). Preparation of acute brain slices using an optimized N‐Methyl‐D‐glucamine protective recovery method. Journal of Visualized Experiments, 00(132), e53825. 10.3791/53825 PMC5931343 29553547

